# The Quarantine Convention of the South Atlantic and Gulf States, at Mobile, Alabama*Condensed from Report in *Philadelphia Medical Journal*.

**Published:** 1898-03

**Authors:** 


### SOCIETY REPORTS.

---

### THE QUARANTINE CONVENTION OF THE SOUTH ATLANTIC AND GULF STATES, AT MOBILE, ALA.*

The convention met Wednesday, February 9th, at 10.40 A.M., and, after the usual formalities of opening, Governor Johnston, of Alabama, was chosen temporary chairman. The convention by vote proceeded to change the program, taking up first the questions of State and National Quarantine. Hon. Hannis Taylor then read a paper on "Quarantine with Reference to International Rights and Interests." The sources whence we receive yellow fever infection are from Brazilian, West Indian, and Peruvian ports; mostly from Havana and Rio de Janeiro. The great difficulty at once encountered is that international law is defective, each nation's system being different and not to be interfered with by outsiders. Only by international courtesy can a common action and remedy be provided. A Quarantine Union, like that of the Postal Union, is necessary, and an agreement upon a uniform system of rules.

Hon. Edward Farrar read a paper upon "Federal and State Powers as to Quarantine," which proved to be a vigorous argument for the States' rights party. Quarantine laws, he said, are health laws and belong to the police power, which has never been taken from the State, and in which rests the protection of the health, life, well-being, and prosperity of the people. He held that if the State did not pass laws to prohibit nuisances, the spread of disinfection, the punishment of incendiarism, murder, assaults, etc., Congress cannot pass such laws. He cited a number of authorities to show that the police powers of the State had never been delegated to the general government. He held that as quarantine laws had been classed as part of the police powers of the State, and that such police power had never been delegated to the Con-

*Condensed from Report in *Philadelphia Medical Journal.*

gress, therefore there was no power, implied or expressed, in Congress to pass quarantine laws. If quarantine laws control commerce between States and nations, Congress can deal with them; but if they simply affect commerce, than the Congress has nothing to do with them, especially if they are *bona fide* health laws; I do not mean shotgun quarantine, nor those which prevent a train from passing from one locality through another. I believe that the enactment of quarantine laws is therefore reserved to the States.

Hon. R. H. Clarke, in the discussion, said: This is a convention gathered for suggesting the best means of keeping yellow fever out of this country. No sooner does an epidemic of yellow fever break out than we begin to call on the government for aid. We want money, camps, rations, and other things, but still we say to it "hands off." Such a state of things can scarcely exist. There are two divisions of quarantine. One is a boundary quarantine, and the question is, has the general government the right to say to a vessel that it shall not enter any port or proceed up any river or harbor. The government has the power to turn back from any harbor of this country men and merchandise. He argued that the general government had the power to do these things.

Dr. S. A. Robinson, of New York, thought that quarantine, or disease-limiting and preventing authority, must be uniform in order to be most effective. Not uniform in methods, for conditions vary and methods must meet conditions, but uniform in spirit and power. The idea that forty-five systems of quarantine (one for each State) could best protect the people of the United States seems preposterous. To thoroughly protect our entire population, such authority and regulations must be national. In proof of this he read an opinion of W. B. Hornblower, of New York, in answer to a request of the New York Board of Trade and Transportation, the summary of which is as follows:

"Conceding the right of the government to interfere with the liberty of the citizen and with his rights of property, so far as may be necessary for the protection of the public health, the only question is, whether it is more expedient that this right should be exercised by the local government or by the State government than by the general government? It seems to me that a uniform system administered by national officers is the only safe and rational method of administering this branch of government. Of course, the tremendous powers imposed upon quarantine officers are open to abuse, but such abuse by national officers would have a wider publicity and be subject to more direct remedial legislation than would the petty tyranny of the local officers, especially where the sufferers from such tyranny would usually be non-residents of the locality where the officers exercised their authority. As quarantine is, of necessity, an interference with commerce, it seems to me that it is peculiarly a matter within the province of Federal legislation."

Mr. John B. Knox of Anniston, also made a telling speech against the States' rights doctrine, and quoted two cases decided by the Supreme Court of the United States, proving the constitutionality of Federal control.

Hon. P. W. Meldrim of Savannah, Ga., then read a paper on "Quarantine and Commerce from a Legal Standpoint." He said the question could be divided into two heads. Has Congress the power to pass an exclusive quarantine law, and, if so, would the passage of such a law be proper? He traced the history of quarantine from the time of the fourteenth century, when Venice quarantined against the plague, down to the present time, speaking of the enactments of the Colonial Congress. He admitted that every quarantine regulation is a restriction upon commerce; but even that restriction has been a protection to the lives of the people. He declared that quarantine is based on the law of nature. He cited the possibility of State and Federal statutes on the subject of quarantine—two laws on the same subject, so that the question to be decided is, which law shall yield. If Congress legislates on this subject, then the State law is abrogated. The public health comes within the strict meaning of the police power, and in this the State is sovereign. He deprecated the drift toward centralization and paternalism.

The second day's session began at 9:30 A.M., with a paper on "Quarantine as it Affects Personal Rights," by Professor G. D. Shands, of the Law Department of the University of Mississippi. He cited opinions that quarantine laws were constitutional when the necessity for the enforcement of the provisions abridging personal rights was apparent and necessary to protect the public health and property. One of these opinions was by the Supreme Court of New York, in effect that before a person's liberty could be infringed that person must either be infected or have been exposed to infection. Another opinion was that in all doubtful cases the decision of the court should lean toward the police power. He held the act must be convenient and appropriate to promote the public health, then the law will be upheld by the courts.

In the discussion, Hon. E. G. Bromberg of Mobile, said the remedy for all the mischievous features of our quarantine is to be found in the education of the people. Ultimately the United States Supreme Court will have to pass upon any rule touching the commerce laws, and so the State Supreme Courts will have to pass upon the local laws. We need a department of health, with its great laboratories, to disseminate information relative to disease, just as the Department of Agriculture disseminates information as to the raising of crops.

Dr. W. H. Sanders, State Health Officer of Alabama, read a paper on "National, State, and Local Quarantines—How Best to Adjust their Differences." He said there are three monarchs in this country—the citizen in his inalienable rights on his own domain; the State in its reserved rights, and the nation in its granted rights. He urged that there must be harmony between the local, State and national quarantine laws, so that they might be enforced with the least friction. Make the State supreme in her own borders, and the problem is largely solved. The function of the general government is to aid and cooperate with the State authorities. He said the system of quarantine he seeks to establish contemplates a national bureau of public health, in which all the States would have a voice, and all the States having equal rights in making the rules and regulations.

The plan contemplates in each county a health officer, backed by a board of health. He would have the health officer one in fact, trained for his work, and have him study the topography of his county, the railroads and county roads through it, and the manner of travel and character of commerce, and would have him study the health laws. He would have a State health officer of the same sort, and would expect him to familiarize himself with the railroad stations in the State; have him know one or two prominent men at each station, so he could call them to his aid by telegraph. Suppose we had a quarantinable disease under such conditions, within an hour there could be a cordon of health officers around the place where that case was found. He would have all public roads guarded, and all trains stopped at the State line till they could be inspected, and would have an officer on the trains to inspect them. Thus there would be a cordon on the borders of the State, and people inside that cordon could transact their business without disturbance by quarantine officers. He would forbid interference with trains by local authorities and lodge this power in the State. He would concede to local authorities to decline to receive freight which they feared would infect their towns, but beyond this they should not be allowed to go.

He had heard many proclaim they favored national quarantine, when three questions would put them on the other side. "If those who urge national quarantine mean that all State lines are to be obliterated, and the country become as one great country, with a horde of quarantine officers receiving their power from a central head far removed from the seat of action, I say, God forbid. It is unnecessary for me to say that we would find ourselves overrun with an ignorant, insolent class, that would lead to bloodshed and disorder."

Dr. F. C. Zacharie read a paper upon the same subject, advising that Congress declare what is a reasonable quarantine, what a reasonable detention, what goods shall be disinfected, and then prescribe a penalty. Let the State pass general laws for the regulation of their State quarantine. He believed such statutes could be carried out.

At the night session Dr. Wingate of Milwaukee, read a paper on "A National Bureau of Public Health." He said that as far as a cabinet officer of health is concerned, he is satisfied that is out of order; but the national bureau can be established without such a cabinet officer. He opposed the enlargement of the powers of the Marine Hospital Service in order to cover a national health bureau.

The time is coming when quarantine will be a relic of barbarism. He referred to a bill on the subject, and said it would give a national bureau of health other powers than those pertaining to quarantine. He believed the States should manage their own quarantines, and the general government should assist, and laws should be adopted so that both would move smoothly.

Dr. C. M. Drake of Atlanta, then read a paper on "National Quarantine." He expressed his surprise at the references he had heard against the Federal government on the floor of the convention. He said that if the recent epidemic proved anything, it proved that the State and local boards of health were unable to prevent the spread of the infection. Their methods were absurd, and often barbarous; one State quarantined against another State, and one city against another, without reason, and still the fever spread. Bridges were burned, mails were stopped, commerce was paralyzed. He spoke of Montgomery's action relative to receiving the mails. The people are disgusted with our present quarantine laws, and they want a change. The States along the Gulf and Atlantic coasts cry out for a change, and that change ought to be known to every thinking man.

Dr. H. B. Horlbeck of Charleston, on the same subject, said that Congress should authorize a "Quarantine Council," one member from each State, to make all rules and regulations as to interstate quarantine, that such regulation shall be referred by the State boards, and, in their failure, by the general government.

The morning session, February 11th, was taken up with a discussion over the majority report of the Committee on Resolutions, and a substitute offered by Hon. R. H. Clarke of Mobile. The finally-reamended Clarke substitute, after a long discussion, was passed at the afternoon session; it is as follows:

*Resolved*, That it is the sense of this convention:

1. That Congress be requested to provide for a department of health as soon as practicable.

2. That it is the sense of this convention that Congress should enact laws to provide for an efficient maritime quarantine, to be uniform and impartial in its application to the different commercial ports of this country, so as to give no one or more of them undue commercial advantage over the others, and to be enforced by the several State and municipal quarantine or health boards, if they will undertake so to do, leaving also to the States the power to prescribe and enforce additional reasonable safeguards of the health of their communities, provided that such State action shall not unreasonably obstruct commerce.

3. That Congress should aid the several States in establishing and maintaining uniform, reasonable, and efficient quarantine laws for effecting, but not regulating, interstate commerce, leaving to each State adequate power to protect as it shall deem best the lives and health of its people.

4. That Congress should leave exclusively to the States the regulation of their purely internal commerce and the provision of such quarantine and sanitary laws and regulations as they may deem advisable to that end.

5. That in the framing of quarantine laws and regulations and in their enforcement Congress should avail itself of the learning, experience and ability of the medical profession in the fullest measure possible, and especially by way of an advisory council.

A paper was then read by Dr. S. R. Olliphant on "Sanitary Inspection of Foreign Ports," and a number of papers were passed to the secretary for publication.

An interesting paper on "Depopulating Cities Infected with Yellow Fever, and How Best to Accomplish It," was read by Dr. Felix Formento of New Orleans. The following were the conclusions:

1. General depopulating of towns and cities infected with yellow fever, however desirable, is not practicable.

2. Voluntary exodus of individuals and families is to be encouraged at the first breaking out of the disease.

3. During an epidemic, depopulating should be carried out under special sanitary precautions.

4. Depopulating of houses in which first cases occur, by removal of the sick to properly constructed hospitals, and of the well to special camps of observation, and thorough disinfection of houses, we consider the most efficient measure for the stamping out of the disease at its birth.

5. Isolation of the sick and destruction or disinfection of discharges from bowels, and of all contaminated material, with disinfection of the premises, are the best means of limiting the disease.

6. All measures of repression of an arbitrary character are to be applied to a limited number of cases only. If they fail, they should be discontinued, as they are an additional burden to the sufferings and hardships of the people.

---

### HYPODERMOCLYSIS.

The subject of subcutaneous injection of salt solution is discussed by Dr. George S. Brown, of Birmingham, Ala., in a late issue of the *New York Medical Journal.* After detailing a number of surgical cases, in which the treatment apparently saved life, he says: "I feel confident that from time to time in the near future we shall have encouraging reports of the use of hypodermoclysis in the treatment of many diseases in which it has not as yet been used. It undoubtedly increases the number and activity of the leucocytes. By increasing the arterial pressure it also increases the action of the skin and kidneys. Increased leucocytosis and increased elimination are the conditions most to be desired in combating toxemias. Reasoning from the ground of its diluent and eliminative effects, hypodermoclysis ought to give good results in the treatment of cases of typhoid fever. In those cases in which the active symptoms of the disease have disappeared and the patient is left in a prostrated condition, with a slight daily fever which he seems unable to throw off, it ought to be of the greatest value by its diluent and eliminating action. Indeed all through the active stage of the disease it might be of benefit as a stimulant. By its diluent and eliminative action it might be of value in the treatment of acute rheumatism, and, indeed, in all that class of toxemias which react so violently on the nervous system—*e. g.*, in the late stages of severe cases of diphtheria and scarlet fever it might save life, as it has done in so many surgical conditions, septicemia, shock, excessive hemorrhage, etc.—*American Journal of Medicine and Surgery.*

